# The complex clinical response to selective serotonin reuptake inhibitors in depression: a network perspective

**DOI:** 10.1038/s41398-022-02285-2

**Published:** 2023-01-21

**Authors:** Lynn Boschloo, Fredrik Hieronymus, Alexander Lisinski, Pim Cuijpers, Elias Eriksson

**Affiliations:** 1 Department of Clinical Psychology, Utrecht University, Utrecht, The Netherlands; 2 Department of Clinical, Neuro, and Developmental Psychology, Amsterdam Public Health Research Institute, Vrije Universiteit Amsterdam, Amsterdam, The Netherlands; 3 Department of Pharmacology, Institute of Neuroscience and Physiology, Sahlgrenska Academy, University of Gothenburg, Gothenburg, Sweden; 4 International Institute for Psychotherapy, Babeș-Bolyai University, Cluj-Napoca, Romania

**Keywords:** Depression, Clinical pharmacology

## Abstract

The clinical response to selective serotonin reuptake inhibitors (SSRIs) in depression takes weeks to be fully developed and is still not entirely understood. This study aimed to determine the direct and indirect effects of SSRIs relative to a placebo control condition on clinical symptoms of depression. We included data of 8262 adult patients with major depression participating in 28 industry-sponsored US Food and Drug Administration (FDA) registered trials on the efficacy of SSRIs. Clinical symptoms of depression were assessed by the 17 separate items of the Hamilton Depression Rating Scale (HDRS) after 1, 2, 3, 4 and 6 weeks of treatment. Network estimation techniques showed that SSRIs had quick and strong direct effects on the two affective symptoms, i.e., depressed mood and psychic anxiety; direct effects on other symptoms were weak or absent. Substantial indirect effects were found for all four cognitive symptoms, which showed larger reductions in the SSRI condition but mainly in patients reporting larger reductions in depressed mood. Smaller indirect effects were found for two arousal/somatic symptoms via the direct effect on psychic anxiety. Both direct and indirect effects on sleep problems and most arousal/somatic symptoms were weak or absent. In conclusion, our study revealed that SSRIs primarily caused reductions in affective symptoms, which were related to reductions in mainly cognitive symptoms and some specific arousal/somatic symptoms. The results can contribute to disclosing the mechanisms of action of SSRIs, and has the potential to facilitate early detection of responders and non-responders in clinical practice.

## Introduction

The clinical response to selective serotonin reuptake inhibitors (SSRIs) in depression takes weeks to be fully developed [1]. While it has been suggested that initial improvements in mood, or negative biases in emotional processing [2], may precede later improvements in other domains, empirical evidence remains limited. Shedding further light on this issue may aid attempts to disclose the mechanisms of action of these drugs, and may also facilitate early detection of responders and non-responders in clinical practice.

A recent comprehensive post hoc analysis [3] showed substantial differences in the response of individual symptoms to SSRIs relative to placebo, the largest effects being found for two affective symptoms: depressed mood (standardized mean difference = −0.40) and psychic anxiety (standardized mean difference = −0.30). Substantial effects were also found for symptoms that may be regarded as cognitive symptoms (e.g., feelings of guilt or loss of interest in work/activities), whereas effects on other types of symptoms (e.g., arousal/somatic symptoms and sleep problems) were in general much smaller, absent or even negative. These findings suggest that a focus on individual symptoms results in a more nuanced assessment of treatment efficacy and also has potential in improving our understanding of the chain of events leading to a clinical response to SSRIs.

An interesting next step would be to focus on the interrelatedness of clinical symptoms. In the past decade, network estimation techniques have shown to be valuable in unraveling the complex relations between symptoms [4–6], also before and after treatment [7, 8], and in revealing the complex relations between symptom-specific changes during treatment [9–14]. In a recent study on the efficacy of SSRIs relative to cognitive behavioral therapy [14], we used network estimation techniques to reveal the relationships of symptom-specific changes during treatment as assessed with the 17 individual items of the Hamilton Depression Rating Scale (HDRS [15]). Consequently, we could distinguish symptom-specific effects of treatment that were independent of the effects on other symptoms (i.e., direct effects) versus effects that could be explained by effects on other symptoms (i.e., indirect effects).

SSRIs were directly related to larger reductions in the two considered affective symptoms (i.e., depressed mood and psychic anxiety), which were related to considerable reductions in specific other -mainly cognitive- symptoms [14]. Although this suggests a pathway in which SSRIs mainly impact mood, it is important to note that we only considered pre- and post-treatment (i.e., after 8–16 weeks) scores of symptomatology. An important step forward would be to also consider the progression of symptom changes *during* the treatment.

This will be the first study that uses network estimation techniques to shed light on the clinical response to SSRIs relative to placebo by considering individual symptoms after 1, 2, 3, 4 and 6 weeks of treatment. For this purpose, we used individual patient data from 28 industry-sponsored placebo-controlled SSRI trials as previously described [3, 15]. We considered all 17 items of the HDRS [16], comprising a wide range of clinical symptoms, and explored the direct and indirect symptom-specific effects of these drugs at all five follow-up assessments.

## Methods

### Study design

We requested patient-level data for all industry-sponsored, US Food and Drug Administration- (FDA) registered, placebo-controlled, acute-phase, and HDRS-based trials of adults with major depression regarding citalopram from Lundbeck (Valby, Denmark), regarding paroxetine from GlaxoSmithKline (Brentford, UK), and regarding sertraline from Pfizer (New York, NY, USA). We obtained data from all relevant studies except for three small, prematurely terminated trials: GSK/07 (*n* = 25), LB/86 A (*n* = 24), and LB/87 A (*n* = 34). We also included ten post-registration or post-marketing trials as provided by GlaxoSmithKline and Pfizer. In three studies (GSK/115, GSK/128, and PZ/111), the active-control groups received fluoxetine; these patients were included in all analyses but those treated with an active comparator other than an SSRI were not. Detailed information on this data set has already been provided [15].

In total, we used data from 8262 patients with major depression who participated in 28 SSRI trials. Participants were treated with either citalopram (*n* = 744), paroxetine (*n* = 2981), sertraline (*n* = 1202), fluoxetine (active-control group; *n* = 754), or placebo (*n* = 2581). Of the 8262 included participants, 8255 (99.9%) had complete symptom data at the pretreatment assessment. Of these 8255 patients, 7909 (95.8%) had complete symptom data at one or more follow-up assessments and comprised the sample for our analyses. It is however important to note that the numbers of participants differed across the different assessments (i.e., week 1: *N* = 7536, 91.3%; week 2: *N* = 6813, 82.5%; week 3: *N* = 5703, 69.1%; week 4: *N* = 6212, 75.3%; week 6: *N* = 4866, 58.9%), but all resulting in sufficient power for the network estimations.

### Assessment of clinical symptoms

Individual clinical symptoms were assessed by the separate items of the 17-item HDRS [16], both before treatment and at assessments after 1, 2, 3, 4 and 6 weeks of treatment. To ease the interpretation of the estimated symptom networks and in line with our previous study [14], the 17 symptoms were divided into five categories: two symptoms that may be categorized as affective (depressed mood and psychic anxiety), four that may be regarded as cognitive (feelings of guilt, suicidal thoughts, loss of interest in work/activities and retardation including concentration difficulties), seven that are related to arousal and bodily functions (agitation, somatic anxiety, general somatic symptoms including lack of energy, genital symptoms, hypochondriasis, and gastrointestinal symptoms), three related to sleep (early night, middle night, and early morning insomnia), and one concerning lack of insight. Items are scored from either 0 to 4 (all affective and cognitive symptoms, the arousal/somatic symptoms of somatic anxiety, and hypochondriasis) or 0 to 2 (most arousal/somatic symptoms, all sleep symptoms, and lack of insight).

### Statistical analyses

First, baseline characteristics were compared between the treatment conditions using *Χ*^2^ statistics for categorical variables (i.e., gender) and independent samples *t*-tests for continuous variables (i.e., age and the 17 individual symptom scores). Given the large number of tests on individual symptoms, Bonferroni correction was applied and, consequently, the statistical significance value was set at *p* = 0.05/17 = 0.003.

All network estimations were performed using R (version 3.6.2). First, we examined the *direct* and *indirect* effects of SSRIs on individual symptoms at the 1-week follow-up assessment. For this purpose, a network including treatment condition (binary variable) and individual symptoms (continuous variables) was estimated with package *mgm* [17] using a mixed graphical model. In the network model, LASSO regularization was applied using cross-validation (CV; 10 folds) to select the optimal tuning parameter. Package *qgraph* [18] was used to visualize the network, with a fixed layout in which symptoms of the same category are placed together. The resulting network shows the complex response of SSRIs; direct connections of treatment condition with individual symptoms can be considered as direct treatment effects, whereas the connections of symptoms to other symptoms can be considered as indirect treatment effects. To explore how the network develops over time, we used the same approach to estimate separate networks including treatment condition and symptom scores at the 2, 3, 4, and 6 weeks of treatment.

We also focused on the *overall effects* (i.e., not adjusted for other symptom-specific effects) of SSRIs on individual symptoms by performing independent sample t-tests comparing symptom scores between treatment conditions and calculating corresponding effect sizes (i.e., Cohen’s d) at the all assessments. To explore whether differences in symptom scores were a consequence of direct effects, indirect effects, or both, we compared these overall effects with the outcomes of the network estimation techniques identifying direct effects of SSRIs.

Lastly, we performed a set of sensitivity analyses. To evaluate the edge weight accuracy of the network models (at week 1, 2, 3, 4 and 6), we used the *resample* function as implemented in *mgm* [17]. For each model, we ran one hundred bootstrap samples for which we fitted the model, and reported the resulting sampling distribution of the most relevant edges (i.e., the direct treatment effects). Secondly, we explored the potential confounding effects of the included trials and baseline characteristics (i.e., gender and age). For this purpose, we estimated a network including treatment condition, individual symptoms at week 6 as well as trial-id and, separately, any baseline characteristic that was significantly related to treatment condition. We explored whether the resulting networks differed from the network including only treatment condition and individual symptoms at week 6.

## Results

### Baseline characteristics

Participants with complete data at one or more post-assessments (*N* = 7909) did not differ from participants with incomplete data at all post-assessments (*N* = 346) in any of the seventeen clinical symptoms at baseline, but displayed small differences with respect to gender (female: 59.5% versus 63.3%, *p* < 0.001) and age (44.3 versus 40.1 years, *p* < 0.001). Of the 7909 participants who had complete post-assessment symptom data at one or more follow-up assessments, 5424 received SSRIs and 2485 placebo. No significant differences between conditions were found for gender or individual symptoms at baseline, but there was a small difference in age (mean 45.1 years, sd 14.8, for the placebo condition versus mean 43.9 years, sd 14.4, for the SSRI condition, *p* = 0.001; Table 1).

**Table 1:** Baseline characteristics.

	Placebo (*N* = 2485)	SSRIs (*N* = 5424)	*p*
Female gender, No. (%)	1469 (59.1)	3234 (59.6)	0.67
Age in years, mean (SD)	45.1 (14.8)	43.9 (14.4)	0.001
Individual depressive symptoms			
*Affective symptoms*			
Depressed mood, mean (SD)	2.8 (0.6)	2.8 (0.6)	0.07
Psychic anxiety, mean (SD)	2.2 (0.7)	2.2 (0.8)	0.86
*Cognitive symptoms*			
Feelings of guilt, mean (SD)	1.7 (0.7)	1.7 (0.7)	0.96
Suicidal thoughts, mean (SD)	1.1 (0.9)	1.1 (0.9)	0.36
Loss of interest in work/activities, mean (SD)	2.7 (0.6)	2.8 (0.6)	0.02
Retardation/concentration problems, mean (SD)	1.1 (0.8)	1.1 (0.8)	0.42
*Arousal/somatic symptoms*			
Agitation, mean (SD)	1.1 (0.8)	1.1 (0.9)	0.41
Somatic anxiety, mean (SD)	1.6 (0.8)	1.6 (0.8)	0.34
General somatic symptom, mean (SD)	1.7 (0.5)	1.7 (0.5)	0.17
Genital symptom, mean (SD)	1.3 (0.8)	1.3 (0.8)	0.84
Hypochondriasis, mean (SD)	0.9 (0.8)	0.9 (0.8)	0.22
Gastrointestinal symptoms, mean (SD)	0.6 (0.7)	0.6 (0.7)	0.03
Loss of weight, mean (SD)	0.3 (0.6)	0.3 (0.6)	0.39
*Sleep problems*			
Early night insomnia, mean (SD)	1.2 (0.8)	1.2 (0.8)	0.69
Middle night insomnia, mean (SD)	1.4 (0.7)	1.3 (0.8)	0.005
Early morning insomnia, mean (SD)	1.2 (0.8)	1.2 (0.8)	0.02
*Insight*			
Lack of insight, mean (SD)	0.2 (0.4)	0.2 (0.4)	0.46

### The direct and indirect symptom-specific effects of SSRIs

**Fig. 1:**
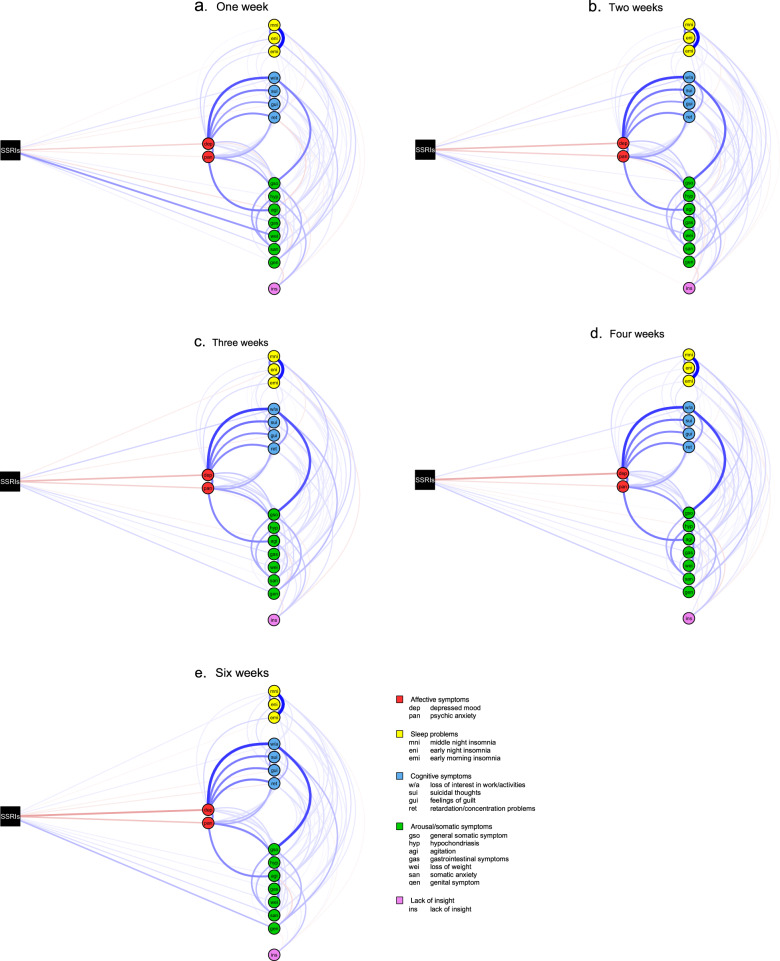
Direct and indirect effects of SSRIs on 17 individual depression symptoms during six weeks of treatment. **a** Effects after 1 week of treatment. **b** Effects after 2 weeks of treatment. **c** Effects after 3 weeks of treatment. **d** Effects after 4 weeks of treatment. **e** Effects after 5 weeks of treatment. Treatment (SSRIs over placebo) is represented by a square and individual symptoms by circles. Red edges between SSRIs and symptoms indicate beneficial treatment effects (i.e., lower symptom scores for the SSRI condition relative to placebo), whereas blue edges indicate detrimental treatment effects (i.e., higher symptom scores for the SSRI condition relative to placebo). Red edges between symptoms indicate negative associations, whereas blue edges indicate positive associations. Thicker edges represent stronger connections.

**Fig. 2:**
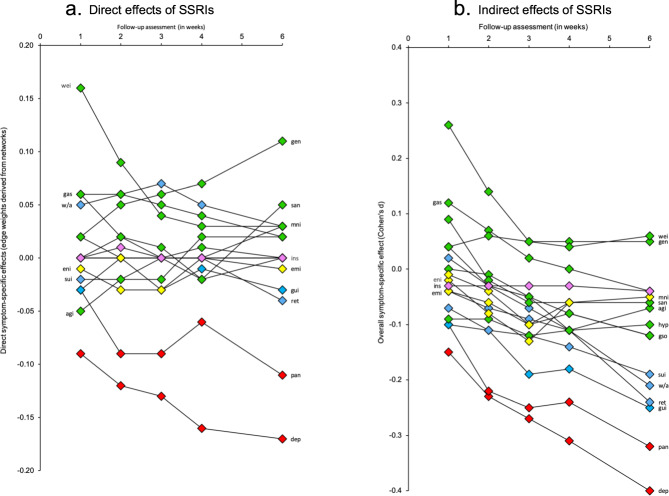
The development of symptom-specific effects of SSRIs during six weeks of treatment. **a** Direct effects of SSRIs. **b** Overall effects of SSRIs.

To explore how the clinical response to SSRIs progressed over time, we estimated separate networks including treatment condition and symptom scores at 1, 2, 3, 4, and 6 weeks of treatment (see Fig. 1). Derived from these networks, Fig. 2 (panel a) specifically presents the magnitude of the direct symptom-specific effects of SSRIs at each of the assessments. Interestingly, these direct symptom-specific effects remained rather stable over time; i.e., the edge weights were strongly correlated across assessments (i.e., week 1–2: *r* = 0.84; week 2–3: *r* = 0.94; week 3–4: *r* = 0.92; week 4–6: *r* = 0.89).

#### Direct effects

At almost all assessments, the strongest direct beneficial effects of SSRIs were found for the two affective symptoms, i.e., depressed mood (e.g., edge weight = −0.17 at week 6) and psychic anxiety (e.g., edge weight = −0.11 at week 6). The effect on depressed mood was already substantial at week 1 (i.e., edge weight = −0.09) and the effect on psychic anxiety at week 2 (edge weight = −0.09), and both became stronger in the following weeks. Other direct beneficial effects were much weaker at all assessment (i.e., edge weights ≥ −0.05 for all symptoms at all assessments).

Interestingly, we also found some detrimental effects of active treatment. The detrimental effect of SSRIs on genital problems gradually increased over time, with an edge weight of 0.02 at week 1 and of 0.11 at week 6. The direct aggravating effect of SSRIs on loss of weight was initially high (i.e., edge weight of 0.16 at week 1) and decreased over time (i.e., edge weight of 0.03 at week 6). Other direct detrimental effects were much weaker (i.e., edge weights ≤ 0.06 for all other symptoms at all assessments).

#### Indirect effects

Figure 1 also shows that symptoms were related in an intricate way, illustrating the complexity of the clinical response to SSRIs. As the strongest direct beneficial effects were consistently found for the two affective symptoms, we zoomed in on the connections of these symptoms with other symptoms (i.e., indirect effects). At all assessments, depressed mood was most strongly connected to psychic anxiety (e.g., edge weight = 0.20 at week 6) and all four cognitive symptoms (e.g., edge weights ranging from 0.20 to 0.31 at week 6). Psychic anxiety showed the strongest connections to depressed mood (e.g., edge weight = 0.20 at week 6), specific arousal/somatic symptoms (e.g., agitation and somatic anxiety; both edge weights = 0.19 at week 6), and cognitive symptoms (e.g., feelings of guilt and loss of interest in work/activities; edge weights = 0.12 and 0.10 at week 6, respectively).

### Overall effects of SSRIs over time

Lastly, we focused on the overall symptom-specific effects of SSRIs at all assessments (i.e., not adjusted for other symptom-specific effects; Fig. 2, panel b) and compared these to the direct effects as derived from the networks (Fig. 2, panel a). At all assessments, SSRIs had the strongest overall effects on depressed mood (e.g., Cohen’s d = −0.40 at week 6) and psychic anxiety (e.g., Cohen’s d = −0.31 at week 6), which is in line with the strong direct effects identified by the network estimations. In contrast, the overall effects of SSRIs on cognitive symptoms were substantial (i.e., Cohen’s d ranging from −0.25 to −0.19 at week 6), whereas the direct effects were small. The detrimental overall effect of SSRIs on loss of weight was largely in line with its direct effect. No overall effect on genital problems was found, whereas the networks revealed a direct detrimental effect of active treatment that gradually became stronger over time.

### Sensitivity analyses

To assess the accuracy of the most relevant edge weights in the estimated networks (Fig. 1), we performed a set of robustness checks. Supplemental Fig. S1 shows the bootstrapped sampling distribution (i.e., 5% and 95% quantiles) of the direct connections of SSRIs (relative to placebo) with depressed mood and psychic anxiety (i.e., the strongest beneficial effects of SSRIs) as well as with genital problems and loss of weight (i.e., the strongest detrimental effects of SSRIs) after 1, 2, 3, 4 and 6 weeks of treatment. The 5% and 95% quantiles did not include zero for depressed mood and -except for the network at week 1- psychic anxiety. The detrimental effect of SSRIs on genital problems gradually increased over time, with the 5% and 95% quantiles including zero at week 1 but not any other week. The direct aggravating effect of SSRIs on loss of weight was initially high and decreased over time, with the 5% and 95% quantiles including zero at week 3, 4 and 6.

To test the robustness of our network findings across trials, we estimated the networks while adjusting for trial-id and found no substantial differences in estimations; at week 6, for example, the beneficial effects of SSRIs on depressed mood (edge weight = −0.17) and psychic anxiety (edge weight = −0.12) and the detrimental effect on genital problems (edge weight = 0.11) were stable. Adjustment for age, which was significantly related to treatment condition (see the previous section on baseline characteristics), did also not substantially change the networks; at week 6, for example, the edge weights of SSRIs with depressed mood (edge weight = −0.17), psychic anxiety (edge weight = −0.11) and genital problems (edge weight = 0.11) remained the same.

## Discussion

To the best of our knowledge, this is the first study that uses network estimation techniques to shed light on the complex clinical response of SSRIs relative to placebo over a 6-week period. The most profound direct effects of SSRIs were found for the two affective symptoms, for which the effect on depressed mood was slightly quicker and stronger than the one on psychic anxiety. Direct effects on other symptoms were weak or absent, except for two detrimental effects on genital problems and loss of weight. We observed substantial indirect effects on all four cognitive symptoms via the direct effect on depressed mood, whereas smaller indirect effects were found for two arousal/somatic symptoms (i.e., somatic anxiety and agitation) via the direct effect on psychic anxiety.

It is well-established from numerous drug trials as well as from clinical experience that the antidepressant effect of SSRIs takes a few weeks to emerge and several weeks to be fully developed [1]. However, when the sum score of different items on a rating scale is used as a measure of response, as is common in clinical trials, useful information regarding the responses of individual symptoms and their interrelatedness may be overlooked (see, for example, the many interesting studies on subsets of symptoms [19–21]). The main finding of the present study is that only two effects of SSRIs, i.e., on depressed mood and on psychic anxiety, appear to be direct, as they could not be explained by any other symptom-specific effects; this is in line with a recent network study on the symptom-specific efficacy of SSRIs relative to cognitive behavioral therapy [14]. These two direct effects on affective symptoms already started in the first weeks and gradually increased during the 6-week period. Interestingly, no substantial direct effects in favor of SSRIs were found for any of the other symptoms at any of the follow-up assessments. This may suggest that primarily improvements in affective symptoms play a central role in the response to SSRI treatment.

To generate hypotheses regarding the potential mechanisms of clinical change during SSRI treatment, we used network estimation techniques to reveal the patterns according to which individual symptoms were related. The networks showed multiple connections (e.g., 75 unique connections at week 1 and 53 unique connections at week 6), illustrating the complexity of the clinical response to SSRIs. It is therefore unlikely that this clinical response is a consequence of a single mechanism; instead, many mechanisms are probably involved, which may also differ across individual patients. However, our network findings could be valuable in revealing pathways that potentially play a prominent role in the clinical response to SSRIs. For example, depressed mood was mainly related to cognitive symptoms at all assessments, which explains the substantially larger reductions in these symptoms in the SSRI condition relative to the placebo condition. From a clinical perspective, it is also intuitive that an improvement in mood increases, for example, a patient’s interest in work and activities and decreases his or her feelings of guilt and suicidal thoughts. Although to a lesser extent, psychic anxiety was mainly related to somatic anxiety and agitation (i.e., two arousal/somatic symptoms), suggesting that improvements in the psychological aspects of anxiety go hand in hand with improvements in physical aspects of anxiety as measured with these two symptoms; again, this makes sense from a clinical perspective.

The presented networks also support the notion that the HDRS captures common side effects of SSRIs, which is in line with a recent study [22]. For example, SSRIs had a direct effect on loss of weight, which was strong at week 1 and gradually weakened over time, which is in line with previous studies showing SSRIs to be associated with weight loss upon short-term use [23] as well as with the well-established appetite-reducing effect of serotonin [24]. We also found a direct effect of SSRIs on genital problems, which became more profound during the 6 weeks of treatment and which is in line with previous studies on SSRI-induced sexual dysfunction [25] as well as with an extensive literature showing serotonin to dampen sexual behavior across species [26]. However, no overall effect on genital problems was found, suggesting an additional pathway acting in the opposite direction by which SSRI treatment, via the effect on, for example, depressed mood, leads to improvements in sexual function which may mask the identified direct effect.

A strength of the current study is that we used data of 7909 patients with major depression by combining 28 industry-sponsored, placebo-controlled SSRI trials and considered assessments after 1, 2, 3, 4 and 6 weeks. Consequently, we had sufficient statistical power to consider a broad spectrum of clinical outcomes and their complex interrelatedness.

As a possible weakness of the study it should be noted that some reports have suggested the inter-rater reliability of some HDRS items to be poor [27, 28]. Although others were more positive [29], more research is needed on the reliability and validity of assessing individual symptoms with separate items of existing depression scales. It is also important to note that any categorization of symptoms—by definition—results in loss of information and that the categorization presently suggested might be overly simplistic; for example, affective symptoms may comprise both a mood (e.g., feeling sad) and a cognitive component (e.g., “everything is hopeless”) and something similar might be true for other symptoms. An interesting next step would be to consider the differential roles of these components in the clinical response to SSRIs, as it, for example, has been hypothesized that SSRIs do not directly target mood but rather the cognitive biases in emotional processing [2].

It is also important to note that regularization techniques in network estimations set very weak connections to zero [17]. Consequently, they are conservative in identifying connections which implies that, in reality, SSRIs may have very weak direct effects on more symptoms than identified in the networks. Network estimations are therefore not intended to formally test for mediation, but do provide insights into the patterns according to which symptoms are related and can be used in generating hypotheses. An important next step in unraveling the actual working mechanisms of SSRIs would be to examine the longitudinal interrelations between symptoms by considering data of multiple assessments with short time intervals (e.g., daily assessments or even more frequently). It would be important to zoom in on the first week(s) of SSRI treatment, as, for example, the direct effects on the affective symptoms were already present after 1/2 weeks.

Another potential weakness of the current study is that the number of response categories of the HDRS items differ and the sensitivity to detect symptom-specific effects might be higher for items with more response categories. This was, however, not supported by our findings, as the strongest direct effects were found for the two affective symptoms (scored 0–4) and two specific arousal/somatic symptoms (scored 0–2) while only weak effects were found for, for example, hypochondriasis (scored 0–4).

In conclusion, our explorative study shows that network estimation techniques are valuable in demonstrating the complexity of the clinical response to treatment and in identifying pathways that potentially play a prominent role herein. For example, we showed that SSRIs primarily resulted in reductions in affective symptoms, which were related to reductions in mainly cognitive symptoms and some arousal/somatic symptoms. This might be an important step in disclosing the mechanisms of action of SSRIs, and may also have potential in the early detection of responders and non-responders in clinical practice.

## Supplementary information

Supplemental Figure S1
