# Supplementary material for: The complex clinical response to selective serotonin reuptake inhibitors in depression: a network perspective
Source: Transl Psychiatry. 2023 Jan 21;13:19. doi: 10.1038/s41398-022-02285-2 (PMC9867733; doi:10.1038/s41398-022-02285-2)

Supplemental Figure S1. Bootstrapped sampling distribution of the most relevant edge weights

Bootstrapped sampling distribution (i.e., 5% and 95% quantiles) of the edge weights of the direct connections of treatment condition (SSRIs relative to placebo) with depressed mood (dep), psychic anxiety (pan), genital problems (gen) and loss of weight (wei) after one, two, three, four and six weeks of treatment.

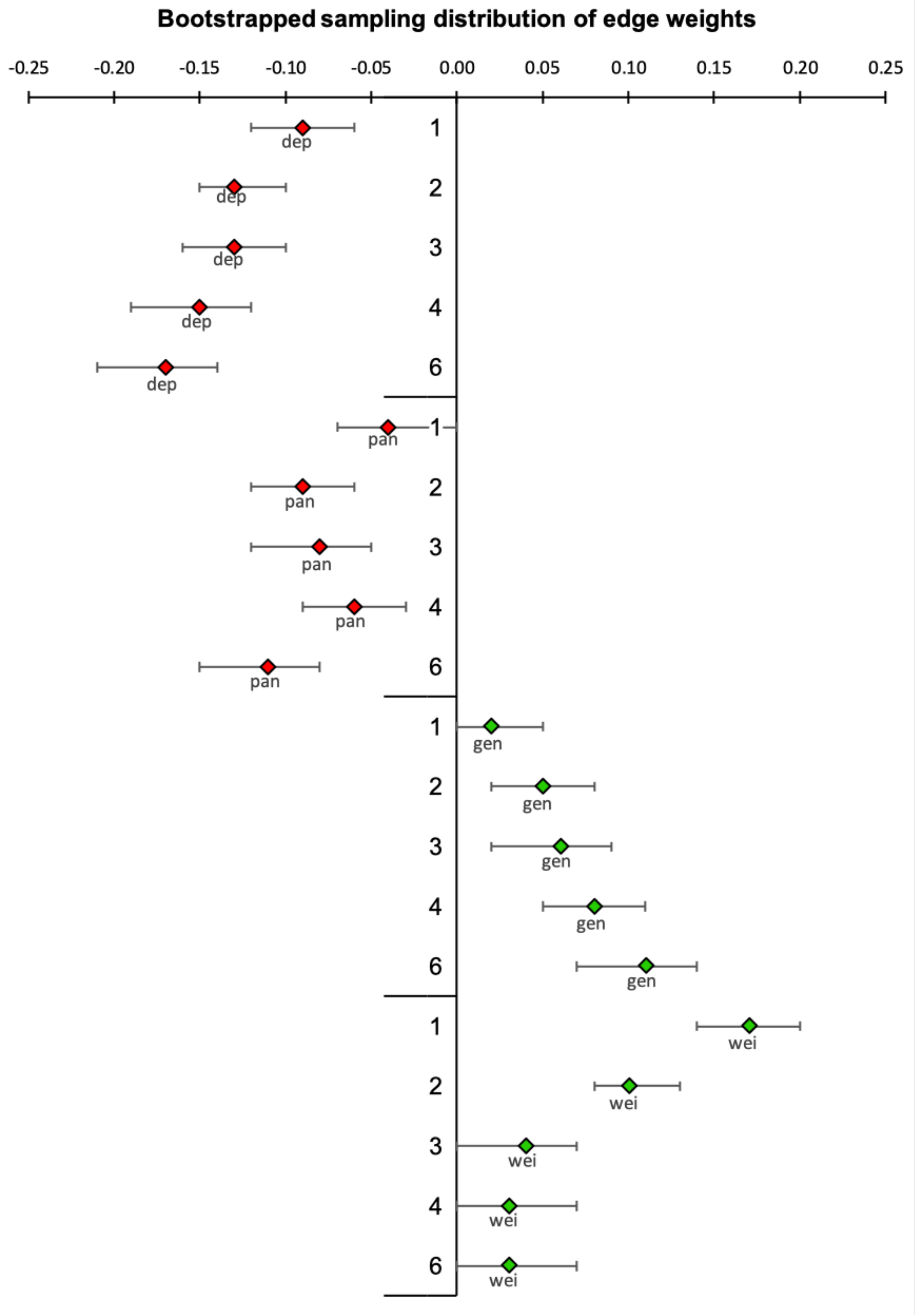

Supplement: Supplementary file 1 — Supplemental Figure S1 [file 41398_2022_2285_MOESM1_ESM.pdf]
